# Andrographolide protects mouse astrocytes against hypoxia injury by promoting autophagy and S100B expression

**DOI:** 10.1590/1414-431X20177061

**Published:** 2018-04-19

**Authors:** Juan Du, Chunyan Zhang, Xueqing Na, Aizhi Li, Qingfeng Zhang, Kezhong Li, Yongbo Ding

**Affiliations:** 1 Department of Anesthesiology, The Affiliated Yantai Yuhuangding Hospital of Qingdao University, Yantai, China; 2 Department of Anesthesiology, The Second Affiliated Hospital of Kunming Medical University, Kunming, China; 3 Department of Anesthesiology, School of Medicine, Shandong University, Jinan, China

**Keywords:** Andrographolide, Hypoxia-injured astrocytes, JNK pathway, S100B, ATG5

## Abstract

Andrographolide (ANDRO) has been studied for its immunomodulation, anti-inflammatory, and neuroprotection effects. Because brain hypoxia is the most common factor of secondary brain injury after traumatic brain injury, we studied the role and possible mechanism of ANDRO in this process using hypoxia-injured astrocytes. Mouse cortical astrocytes C8-D1A (astrocyte type I clone from C57/BL6 strains) were subjected to 3 and 21% of O_2_ for various times (0–12 h) to establish an astrocyte hypoxia injury model *in vitro*. After hypoxia and ANDRO administration, the changes in cell viability and apoptosis were assessed using CCK-8 and flow cytometry. Expression changes in apoptosis-related proteins, autophagy-related proteins, main factors of JNK pathway, ATG5, and S100B were determined by western blot. Hypoxia remarkably damaged C8-D1A cells evidenced by reduction of cell viability and induction of apoptosis. Hypoxia also induced autophagy and overproduction of S100B. ANDRO reduced cell apoptosis and promoted cell autophagy and S100B expression. After ANDRO administration, autophagy-related proteins, S-100B, JNK pathway proteins, and ATG5 were all upregulated, while autophagy-related proteins and s100b were downregulated when the jnk pathway was inhibited or ATG5 was knocked down. ANDRO conferred a survival advantage to hypoxia-injured astrocytes by reducing cell apoptosis and promoting autophagy and s100b expression. Furthermore, the promotion of autophagy and s100b expression by ANDRO was via activation of jnk pathway and regulation of ATG5.

## Introduction

Traumatic brain injury (TBI) is a major cause of death or disability worldwide, especially in young people. After severe TBI, many people are killed by secondary injury, which is a complex set of cellular processes following trauma (1). These secondary processes can worsen the damage and account for the greatest number of TBI deaths (2). Brain hypoxia is a common cause of secondary injury. Brain hypoxia initiates a sequence of biochemical events to induce neuronal cell apoptosis, resulting in a hypoxic brain injury and dysfunction (3). Thus, brain hypoxia can aggravate TBI and is regarded as an independent predictor or a marker of disease severity (4).

*Andrographis paniculata* is a medicinal herb that is widely used in China and other parts of Asia for the treatment of upper respiratory tract infections, fever, and diarrhea (5,6). Andrographolide (ANDRO) is the major active component isolated from the stem and leaves of *A. paniculata* and is a natural diterpenoid lactone (7). ANDRO has been studied for its various bioactivities including immunomodulation (8), anti-inflammatory (9), anti-cancer (10), anti-viral (11), anti-bacterial (12), anti-hyperglycemic (13), and neuroprotective (14). The effect of ANDRO on cell apoptosis is complex. ANDRO protects thymocytes or endothelial cells against apoptosis (15,16). On the other hand, some reports showed that ANDRO could induce apoptosis in human prostatic adenocarcinoma PC-3 cells (17) or other cancer cells (18). In addition, a previous study has showed that ANDRO could protect rat cardiomyocytes against hypoxia and reoxygenation injury (19). Furthermore, ANDRO was reported to protect mice against hypoxia/ischemia-induced oxidative brain injury (20). Therefore, we aimed to investigate whether ANDRO has an effect on mouse hypoxia-injured brain cells, which is still unknown.

Astrocytes are the most widely distributed class of cells in mammalian brain. These cells perform many functions, including support of endothelial cells, fueling neurons, maintenance of extracellular ion balance, nervous repair, and scarring of the brain following traumatic injuries (21–23). Therefore, we constructed a model of hypoxia injury in mouse astrocytes to investigate the role of ANDRO. To achieve this goal, we revealed the role of ANDRO from three aspects: cell apoptosis, autophagy, and S100B expression.

## Material and Methods

### Cell culture

Mouse cortical astrocytes C8-D1A (astrocyte type I clone from C57/BL6 strains), were purchased from the American Type Culture Collection (ATCC, CRL-2541^TM^, USA). Cells were cultured with high glucose Dulbecco’s modified eagle medium (DMEM, Invitrogen, CA) containing 10% fetal bovine serum (FBS, Gibco, USA), 50 IU/mL penicillin G (Gibco, USA) and 50 μg/mL streptomycin (Gibco). Then, cells were incubated at 37°C in a moist atmosphere containing 5% CO_2_.

### Hypoxia exposure

To establish an astrocytes hypoxia injury model, cells were subjected to hypoxic (3% O_2_, 5% CO_2_ balanced with N_2_) culture. Cells cultured in normoxia (room air, 5% CO_2_) were the control.

### ANDRO treatment

ANDRO was purchased from Sigma-Aldrich (365645, USA) with purity ≥98%. A stock solution with a concentration of 15 mM was prepared by dissolving ANDRO in dimethyl sulphoxide (Sigma-Aldrich, USA). ANDRO in a concentration of 10 μM was used to treat cells throughout the hypoxia exposure. SP600125, an inhibitor of JNK, was purchased from Sigma (S5567, USA). SP600125 in a concentration of 10 μM was used to treat cells throughout the hypoxia exposure.

### Viability assay

Cells were seeded on a 96-well plate with 5000 cells/well. Cell viability was measured by the Cell Counting Kit-8 (CCK-8, Dojindo Molecular Technologies, USA). After incubation for 0–12 h at 37°C, cells from control, hypoxia, and hypoxia (12 h)+ANDRO groups were added to CCK-8 solution with 10 μL/well and were incubated for 4 h at 37°C. Then, the absorbance was measured at 450 nM using a Microplate Reader (Bio-Rad, USA).

### Apoptosis assay

Cells for apoptosis detection were washed twice with cold PBS and resuspended in buffer. Then, cells were dyed using Annexin V-FITC/PI apoptosis detection kit (Invitrogen, USA) according to the manufacturer’s instruction. After reaction in dark at room temperature for 10 min, flow cytometry analysis was done by using a FACScan flow cytometer (Beckton Dickinson, USA) to differentiate apoptotic cells (Annexin-V positive and PI negative) from necrotic cells (Annexin-V and PI positive).

### Cell transfection

siRNA targeted against ATG5 (si-ATG5) and non-targeting siRNA (si-NC) used as negative control were synthesized from GenePharma Co. (China). Cell transfections were conducted using Lipofectamine 3000 reagent (Invitrogen, USA) following the manufacturer’s protocol. After 48 h of transfection, cells were collected for further analysis.

### Real-time quantitative reverse transcriptase PCR (qRT-PCR)

Total RNA was isolated from treated cells by using TRIzol reagent (Invitrogen, USA). Reverse transcription was performed by using the Prime Script RT reagent Kit (TaKaRa, China) according to the manufacturer’s instructions. A SYBR Fast qPCR Mix (TaKaRa) was used to quantify the mRNA levels according to the manufacturer’s instructions. β-actin was used as the internal standard. The ^ΔΔ^Ct method was used to calculate changes in expression.

### Western blot

The proteins used for western blot were extracted using RIPA lysis and extraction buffer (Thermo Scientific, USA). The proteins were quantified using the BCA Protein Assay Kit (Thermo Scientific) and were separated by a NativePAGE Novex Bis-Tris Gel system (Invitrogen) according to the manufacturer’s instructions. Then proteins were transferred to a Polyvinylidene Difluoride (PVDF) membrane (Millipore, USA). The membrane was incubated with primary antibodies at 4°C overnight, followed by washing and incubation with secondary antibody marked by horseradish peroxidase at room temperature. Primary antibodies included anti-Bcl-2 (ab59348, 1:1000; Abcam, UK), anti-Bax (ab182733, 1:2000; Abcam), anti-pro-caspase-3 (ab44976, 1:500; Abcam), anti-cleaved-caspase-3 (ab13847, 1:500; Abcam), anti-caspase-9 (ab202068, 1:2000; Abcam), anti-LC3B (ab51520, 1:3000, Abcam), anti-Beclin-1 (ab62557, 1:1000; Abcam), anti-p62 (ab56416, 1:1000; Abcam), anti-β-actin (ab8224, 1:1000; Abcam), anti-S100B (ab52642, 1:1000; Abcam), anti-JNK (ab179461, 1:1000; Abcam), anti-p-JNK (ab124956, 1:5000; Abcam), anti-c-Jun (ab32137, 1:5000; Abcam), anti-p-c-Jun (ab32385, 1:5000; Abcam), and anti-ATG5 (ab108327, 1:5000; Abcam). After adding ECL Plus Western Blotting Substrate (Thermo Scientific) to cover the membrane surface, the signals were captured and the intensity of the bands was quantified using the ChemiDoc™ XRS system (Bio-Rad, USA). β-actin antibody (Abcam) was used as the endogenous protein for reference.

### Statistical analysis

All experiments were repeated three times. The results of multiple experiments are reported as means±SD. Statistical analyses were performed using SPSS 22.0 statistical software (SPSS, USA) using a one-way analysis of variance (ANOVA). A P-value of <0.05 indicates a statistically significant result.

## Results

### Hypoxia affected cell viability, apoptosis, and autophagy of astrocytes

**Figure 1.**
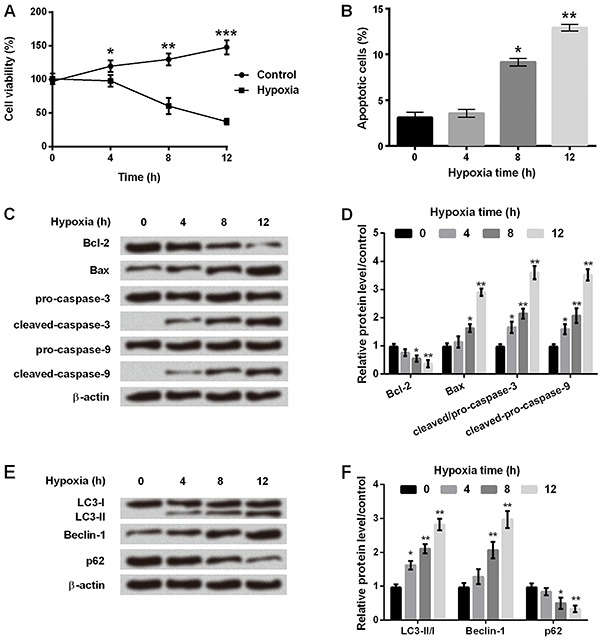
*A*, Cell viability was determined at different times in the control and hypoxia groups. *B*, Apoptosis rate was measured at different times in hypoxic astrocytes. *C*, Protein immunoblots of apoptosis-related factors by western blot assay. *D*, Relative expressions of apoptosis-related factors by quantification of band intensity. *E*, Protein immunoblots of autophagy-related factors by western blot assay. *F*, Relative expressions of autophagy-related factors by quantification of band intensity. β-actin acted as an internal control. Data are reported as means±SD. *P<0.05, **P<0.01, ***P<0.001 compared to 0 h (ANOVA).

After culturing in hypoxia or control (normoxia) environment for 0 to 12 h, C8-D1A cells were harvested and analyzed for cell viability, apoptosis, and autophagy. As shown in Figure 1A, cell viability was significantly reduced at 4 to 12 h (P<0.05, P<0.01 or P<0.001) after hypoxia. Flow cytometry analysis demonstrated that the number of apoptotic cells was significantly increased after 8 h (P<0.05) and 12 h (P<0.01) in hypoxia (Figure 1B). Figure 1C and D showed that Bcl-2 expression was significantly decreased, while Bax, cleaved-caspase-3, and cleaved-caspase-9 expressions were significantly increased after 8 h in hypoxia incubator compared with control (P<0.05 or P<0.01). We also analyzed expression of proteins involved in cell autophagy, and found that the ratio of LC3-II/LC-I and the amount of Beclin-1 were significantly increased, while that of p62 was decreased in a time-dependent manner in hypoxia incubator compared to control (Figure 1E and F, P<0.05 or P<0.01). These results suggested that cell apoptosis and autophagy were significantly increased after 8 h of hypoxic incubation in astrocytes.

### Hypoxia induced S100B expression in astrocytes

**Figure 2.**
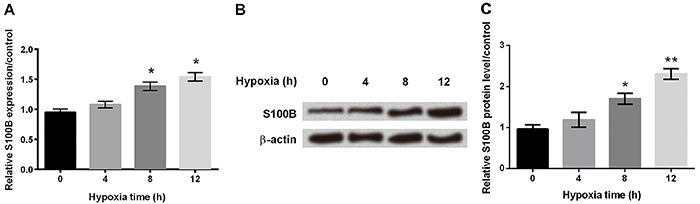
*A*, mRNA expression of S100B was determined by qRT-PCR at different times in hypoxic astrocytes. *B* and *C*, Western blot was performed to assess the protein expression of S100B at different times in hypoxia astrocytes. β-actin acted as an internal control. Data are reported as means±SD. *P<0.05, **P<0.01 compared to 0 h (ANOVA).

Using qRT-PCR, we demonstrated that the mRNA amount of S100B was significantly increased after 8 h in hypoxia (Figure 2A, P<0.05). Western blot also confirmed that protein expression of S100B was gradually increased in hypoxic-treated astrocytes (Figure 2B and C, P<0.05 or P<0.01).

### ANDRO reduced cell apoptosis but promoted cell autophagy in hypoxic astrocytes

**Figure 3.**
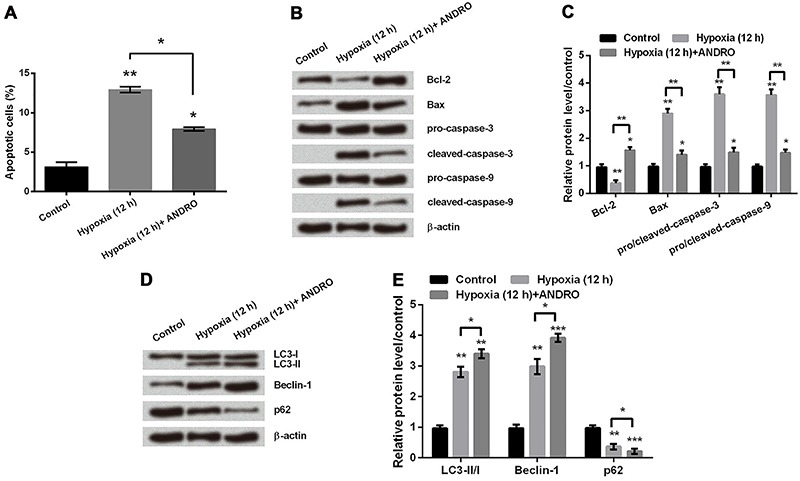
*A*, Apoptosis rate was measured in hypoxic astrocytes with or without treatment of andrographolide (ANDRO). *B* and *C*, Western blot was used to determine the protein expression level of apoptosis related proteins in hypoxic astrocytes with or without treatment of ANDRO. *D* and *E*, Protein expression of autophagy-regulated factors was determined by western blot. β-actin acted as an internal control. Data are reported as means±SD. *P<0.05, **P<0.01, ***P<0.001 (ANOVA).

To reveal the effect of ANDRO on apoptosis, firstly we used flow cytometry analysis. The number of apoptotic cells in hypoxic astrocytes was significantly decreased after adding ANDRO (Figure 3A, P<0.05). Secondly, we detected expression levels of proteins involved in apoptosis by western blot. As shown in Figure 3B and C, addition of ANDRO in hypoxic astrocytes resulted in an increase in Bcl-2 protein expression and decreases in Bax, cleaved-Caspase-3, and cleaved-Caspase-9 protein expressions (P<0.05 or P<0.01). These results suggested that ANDRO attenuates the process of apoptosis in hypoxic astrocytes. By analyzing expression of proteins related to cell autophagy after 12 h in hypoxia, we found that addition of ANDRO significantly promoted increases in LC3-II and Beclin-1 protein expressions, and a decline in p62 protein expression (Figure 3D and E, P<0.05, P<0.01 or P<0.001). This data showed that ANDRO promoted autophagy in hypoxic astrocytes.

### ANDRO promoted upregulation of S100B expression

**Figure 4.**
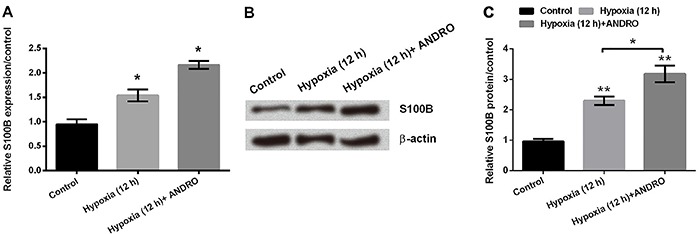
*A*, qRT-PCR was used to determine the mRNA expression of S100B in hypoxic astrocytes with or without treatment of andrographolide (ANDRO). *B* and *C*, Western blot was performed to assess the protein expression of S100B in hypoxic astrocytes with or without treatment of ANDRO. β-actin acted as an internal control. Data are reported as means±SD. *P<0.05, **P<0.01 (ANOVA).

Both qRT-PCR (Figure 4A) and western blot analysis (Figure 4B and C) suggested that addition of ANDRO dramatically increased the expression of S100B in hypoxic astrocytes (P<0.05 or P<0.01).

### ANDRO promoted autophagy and S100B expression by activating JNK signaling pathways

**Figure 5.**
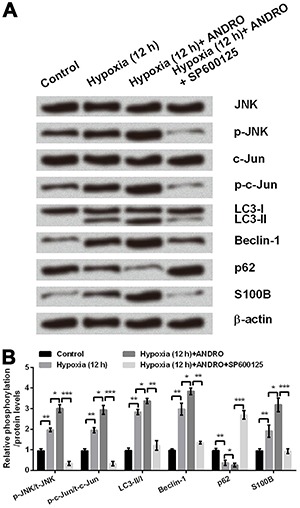
*A*, Protein immunoblots of S100B and proteins related to JNK pathway and autophagy measured by western blot in hypoxic astrocytes with or without treatment of andrographolide (ANDRO) and SP600125. *B*, Relative expressions of JNK pathway and autophagy related factors by quantification of band intensity. β-actin acted as an internal control. Data are reported as means±SD. *P<0.05, **P<0.01, ***P<0.001 (ANOVA).

Next, we investigated the underlying mechanisms through which ANDRO promoted autophagy and expression of S100B. As shown in Figure 5A and B, adding ANDRO alone promoted expressions of S100B and autophagy-related proteins as described above. In addition, ANDRO increased the expressions of p-JNK and p-c-Jun. However, after treatment with JNK inhibitor, ANDRO treatment did not lead to increases in the expressions of LC3-II, Beclin-1, and S100B, or a decline in p62 expression. These results suggested that the promotion of autophagy and S100B expression by ANDRO might be through activating JNK signaling pathways in hypoxic astrocytes.

### ANDRO promoted autophagy and S100B expression by upregulating expression of ATG5

**Figure 6.**
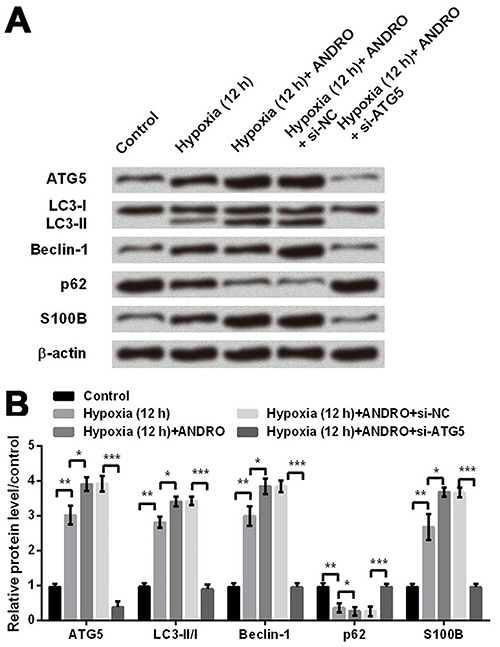
*A*, Protein immunoblots of ATG5, S100B, and autophagy-related proteins measured by western blot in hypoxic astrocytes with or without si-ATG5 transfection and treatment of andrographolide (ANDRO). *B*, Relative expressions of ATG5, S100B and autophagy-related proteins by quantification of band intensity. β-actin acted as an internal control. si-NC: non-targeting siRNA. Data are reported as means±SD. *P<0.05, **P<0.01, ***P<0.001 (ANOVA).

To reveal the regulation mechanism of ANDRO, we also demonstrated the association between ANDRO and expressions of S100B and ATG5. Firstly, we found that expression of ATG5 was increased in hypoxic astrocytes with the treatment of ANDRO (Figure 6A and B, P<0.05, P<0.01 or P<0.001). Given the important role of ATG5 in autophagy process (24), this result provided more evidence that ANDRO promoted autophagy in hypoxic astrocytes. Secondly, when ATG5 was knocked down in hypoxic astrocytes with ANDRO treatment, expression of autophagy-related proteins (LC3-II, Beclin-1, and p62) were reduced, and interestingly the expression level of S100B was also reduced to that in control cells (P<0.05, P<0.01 or P<0.001). Therefore, we concluded that ANDRO promoted S100B expression perhaps by upregulating expression of ATG5.

## Discussion

ANDRO has been well known for its various bioactivities such as anti-inflammatory (9). However, in hypoxia brain injury, the function of ANDRO has not been well studied. Recently ANDRO was reported to protect rat cardiomyocytes against hypoxia and reoxygenation injury and protect mice brain against hypoxia/ischemia injury (19,20). Similarly, in the present study, we demonstrated that ANDRO protected mouse astrocytes against hypoxia injury.

To research the role of ANDRO in hypoxia-injured astrocytes, we investigated its effects on three aspects: cell apoptosis, autophagy, and S100B expression. Flow cytometry analysis was performed to detect apoptotic cell rate, and expressions of a series of apoptosis-related proteins were measured. Caspases play an essential role in the transduction of apoptotic signals. When cytochrome c is released into the cytosol, it binds to an adaptor protein and pro-caspase-9, which in turn cleaves the pro-caspase-9 into the active form (25). Caspase-3 is activated by cleaved-caspase-9 through proteolytic cleavage; then it degrades many intracellular proteins to carry out programed cell death (26). The Bcl-2 family, including anti-apoptotic (Bcl-2, Bcl-XL, Mcl-1) and pro-apoptotic members (Bid, Bax, Bad), is also one of the apoptotic regulatory proteins (27). Therefore, we used cleaved-caspase-3, cleaved-caspase-9, Bcl-2, and Bax as markers of apoptosis in our study. As a result, we found that the apoptotic cell rate and pro-apoptosis-related protein expressions were decreased and anti-apoptosis-related protein expression was increased by ANDRO, indicating that it attenuated the process of apoptosis in hypoxia-injured astrocytes.

Autophagy, also known as type II programmed cell death, causes orderly degradation and recycling of cellular components to survive bioenergetic stress (28). Beclin-1 is a component of the phosphatidylinositol-3-kinase complex that is required for autophagy (29). Beclin-1 expression has been recently used as a marker of autophagy (30,31). During autophagy, there are two ATG5-dependent ubiquitin-like conjugation systems: the ATG12 conjugation system, leading to the formation of ATG12-ATG5-ATG16 molecular complexes, and the LC3 conjugation system, causing the LC3-I to generate a lipidated LC3-II form (24). LC3 also enables the docking of specific cargos and adaptor proteins such as p62 (Sequestosome-1)(32). Both conjugation systems lead to the formation of autophagosomes (24). Therefore, we used Beclin-1, LC3II, and p62 as markers of autophagy in our experiments. Results showed that expressions of Beclin-1 and LC3II were increased and p62 was decreased by ANDRO. Therefore, we concluded that ANDRO promoted autophagy in hypoxia-injured astrocytes. Many studies have shown that autophagy plays a protective role in brain injury (32,33), and our results also confirm this point. Considering that ANDRO inhibited apoptosis while promoting autophagy, we can conclude that ANDRO induced cell autophagy from apoptosis to protect astrocytes from hypoxia damage.

S100B, produced mainly by astrocytes, exerts a neurotrophic effect and was shown to reduce brain injury (34,35). In the present study, we found that the expression of S100B was increased by ANDRO in hypoxia-injured astrocytes. Based on these findings, we inferred that ANDRO protected astrocytes against hypoxia injury by promoting S100B expression.

Next, in order to determine the mechanism responsible for regulation of autophagy and S100B expression by ANDRO, we tested the association between ANDRO, autophagy, and expression levels of S100B, JNK pathway proteins, and ATG5. Activated JNK regulates several important cellular functions including cell growth, differentiation, survival, and apoptosis by activating some small molecules such as c-Jun (36). Recently, JNK has been found to regulate autophagy (37,38). In the current study, we found that ANDRO activated JNK pathway. When JNK pathway was inhibited, ANDRO could not upregulate autophagy and S100B expression as before. These results indicated that the promoting activities of ANDRO on autophagy and S100B expression might be through the JNK pathway. On the other hand, our study showed that knocking down ATG5 resulted in reduction of S100B expression in hypoxia-injured astrocytes with ANDRO treatment. This indicated that ATG5 reduced the expression of S100B even under the action of ANDRO. We, therefore, concluded that ANDRO promoted autophagy via the increased expression of S100B. However, this needs further research.

In conclusion, the present study indicated that ANDRO protected hypoxia-injured astrocytes against apoptosis and promoted autophagy and S100B expression. Our findings may have important implications in the treatment of hypoxia brain injury and application of ANDRO, although further research is still needed.
